# Investigation of total phenolic content and antioxidant activities of *Azadirachta indica* roots

**Published:** 2014

**Authors:** Md. Delowar Hossain, Md. Shahid Sarwar, Syed Masudur Rahman Dewan, Md. Shohel Hossain, AFM Shahid-Ud-Daula, Mohammad Safiqul Islam

**Affiliations:** 1 *Department of Pharmacy, Noakhali Science and Technology University, Sonapur, Noakhali-3814, Bangladesh*

**Keywords:** *Activities DPPH Free Radical*, *Antioxidant*, *Azadirachta indica*, *Scavenging*, *Total Penolic Content*

## Abstract

**Objective**: The present study was an attempt to study total phenolic content and antioxidant property of the crude ethanolic extract of the roots of *Azadirachta indica* (*A. indica*).

**Materials and Methods:** To evaluate the antioxidant properties of the crude extract, some complementary test systems, namely DPPH free radical scavenging assay, reducing power assay, and ferrous ion chelating ability and determination of total phenolic content were conducted.

**Results**: In DPPH free radical scavenging test, IC_50_ value of the crude extract was found to be fairly significant (13.81±0.06 μg/ml) while compared with that of the reference standards, ascorbic acid and BHA (2.12±0.02 and 4.87±0.05 μg/ml, respectively). In reducing power assay, the maximum absorbance for the extract was found to be 1.523±0.026 at100 μg/ml compared with standard ascorbic acid and BHA (2.811±0.013 μg/ml and 2.031±0.019 μg/ml, respectively). The IC_50_ value of the extract as percentage of Fe^++ ^ion chelating ability was determined as 19.01±0.024 μg/ml where EDTA showed 8.87±0.035 μg/ml. The total phenolic amount was also calculated quite high in the extract (238.81±0.98 mg/g of gallic acid equivalent).

**Conclusion: **The assays showed the presence of significant antioxidant properties of the crude sample, which would justify its traditional use. However, it would be very interesting to investigate the possible causes and their mechanisms responsible for the antioxidant property of the plant *A. indica*.

## Introduction

Antioxidants are molecules that are capable of preventing counteracting oxidation of reactive oxygen species (Amin et al., 2013 ▶). Reactive oxygen species (ROS) are generated during cellular metabolism. Oxidative stress, involved in many acute and chronic diseases is mediated by ROS. Thus, the balance between antioxidation and oxidation is believed to be critical to maintain healthy biological systems (Hong and Liu, 2004 ▶). 

Plant sources are rich in antioxidants. Phytochemicals are granting fewer side effects and compatible with the body’s physiology. Therefore, it is the demand of modern era to use such phytoconstituents or phytomedicines (Saikat et al., 2010 ▶). *A. indica*, locally known as *Neem *in Bangladesh*, *belongs to the family Meliaceae. 

It is being used for over 4,500 years in the Indian subcontinent for the treatment of various diseases. It is also used in formulations of Ayurveda. In Ayurveda system, *Neem* is called ‘Sarva Roga Nivarini’ (one that can cure all ailments and illnesses). Almost all parts of the plant including fruits, seeds, leaves, roots, and barks are used in folk remedies (Anon, 1985 ▶). 

Previously, this tree has been screened for free radical scavenging activity and it was found that various parts such as leaves, seeds, flowers, stem bark, and root bark could promote high antioxidant activity (Kiranmai et al., 2011 ▶). Therefore, the present study was conducted to evaluate the antioxidant potential and total phenolic content of the root of *A. indica* to justify its uses in different disease treatments in Bangladesh.

## Materials and Methods

**Plant material collection and identification**

For the present investigation, the roots of *A. indica* were collected by the authors from the surrounding area of Noakhali, Bangladesh, in July, 2011. The plant was identified and authenticated by an expert botanist of Bangladesh National Herbarium (BNH), Mirpur, Dhaka (Accession No. DACB: 32607) and a voucher specimen was submitted at the herbarium for future reference. 

**Extract preparation**

Weighed (400 g of the dried and powdered) sample was soaked in 1.3 L of 80% ethanol (Merck KGaA, Germany) in clean, sterilized, and flat-bottomed glass container. The container with its contents was sealed and maintained for 15 days accompanying occasional stirring and agitation. The complete mixture was then subjected to coarse filtration on a piece of clean, white sterilized cotton material and Whatman^®^ filter paper no. 1. The resulting filtrate was then evaporated on a water bath maintaining 40 ^o^C to dryness and therefore rendered a gummy concentrate of reddish black color. The gummy concentrate was designated as crude extract of ethanol.

**Chemicals used in the antioxidant activity assay**

In the study, 1,1-Diphenyl-2-picryl hydrazyl (DPPH), trichloro acetic acid (TCA), L- ascorbic acid, butylated hydroxy anisole (BHA), gallic acid, Folin-ciocalteu phenol reagent, phosphate buffer (pH 6.6), potassium ferricyanide [K_3_Fe(CN)_6_] (1%), distilled water, EDTA, ferrozine, FeCl_2_, and FeCl_3_ (0.1%) of analytical grade (Merck, Germany) were used for total phenolic content and antioxidant activity assays. 

**DPPH free radical scavenging assay**

 The stable DPPH free-radical scavenging activity was measured using the modified method described by Chang et al. (2001) ▶. Stock solution (1 mg/ml) of the ethanol extract of the roots of *A. indica *was prepared in respective solvent systems from which serial dilutions were carried out to obtain the concentrations of 5, 10, 20, 40, 60, 80, and 100 µg/ml. In this assay, 2 ml of 0.1 mM ethanolic DPPH solution was added to 2 ml of extract solution at different concentrations and the contents were stirred vigorously for 15 sec. Then the solutions were allowed to stand at dark place at room temperature for 30 min occurring chemical reaction. After 30 min, absorbance was measured against a blank at 517 nm with the double beam UV-Visible spectrophotometer. The percentage of DPPH free radical-scavenging activity of plant extract was calculated as:

DPPH free-radical scavenging activity (I %),** = **[(A_0_ – A) /A_0 _] × 100

Where, A_0_ is the absorbance of the control solution (containing all reagents except plant extract); A is the absorbance of the DPPH solution containing plant extract.

The DPPH radical-scavenging activity (%) was plotted against the plant extract concentration (µg/ml) to determine the concentration of extract necessary to decrease DPPH radical-scavenging by 50% (called IC_50_). The IC_50_ value of the extract was estimated by sigmoid non-linear regression, using Sigma Plot 2000 Demo (SPSS Inc., Chicago, IL, USA). All determinations were performed in triplicate. Ascorbic acid was used as positive control standard.

**Reducing power assay**

The method of Dehpour et al. (2009) ▶ was followed to determine the reducing power of *A. indica *roots of ethanolic extract*.* One ml of extract solution of different concentrations (5, 10, 20, 40, 60, 80, 100 μg/ml) was mixed with 2.5 ml of phosphate buffer (0.2 M, pH 6.6) and 2.5 ml of potassium ferricyanide [K_3_Fe(CN)_6_] (1% w/v). The mixture was incubated at 50 °C for 20 min. The reaction was terminated by adding 2.5 ml of trichloroacetic acid (10%, w/v), then the mixture was centrifuged at 3000 rpm for 10 min. The supernatant solution (2.5 ml) was mixed with distilled water (2.5 ml) and ferric chloride (0.5 ml, 0.1% w/v) solution. Then the absorbance was measured at 700 nm against a blank using UV spectrophotometer. Increased absorbance value of the reaction mixture indicates increased reducing power. Three replicates were made for each test sample and average data was noted. Here, ascorbic acid and BHA were used as positive control standard.

**Ferrous ion chelating ability**

The ferrous ions chelating activity of ethanolic extract of *A. indica* and standards were investigated according to the method of Dinis et al. (1994) ▶. Briefly, different concentrations of the extract (5-100 μg/ml) were added to 0.1 ml solution of 2 mM ferrous chloride (FeCl_2_). Then, the reaction was initiated by adding 0.2 ml of 5 mM ferrozine. Then the mixture was shaken vigorously and kept at room temperature for 10 min. After the mixture had reached equilibrium, the absorbance of the solution was then measured at 562 nm in spectrophotometer, wherein the Fe^+2^ chelating ability of extract was monitored by measuring the ferrous ion-ferrozine complex. The percentage of inhibition of ferrozine-Fe^2+^ complex formation was given in the following formula.

Ferrous ions chelating ability (%) **= **[(A_o_ - A) /A_o_] × 100

Where, A_o_ is the absorbance of the control solution (containing all reagents except extract); A is the absorbance in the presence of the sample of plant extract. The test was carried out in triplicate and EDTA was used as standard.

**Investigation of total phenolic content**

**Figure 1:**
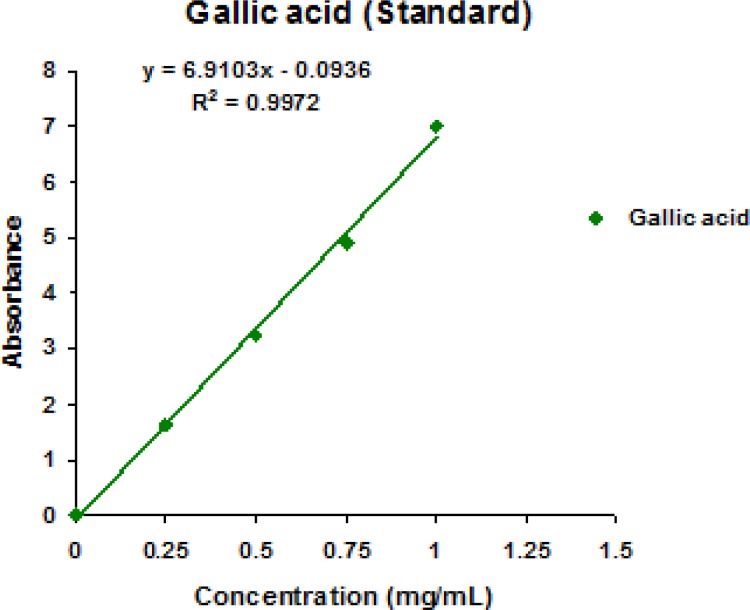
Total Phenolic content of gallic acid (standard).

Using the modified Folin-ciocalteu method, total phenolic content of the extract was determined (Wolfe et al., 2003 ▶). Briefly, 0.5 mL of the extract (1 mg/ml) was mixed with 5 ml Folin-ciocaltu reagent (1:10 v/v distilled water) and 4 ml (75 g/L) of sodium carbonate. Then, the mixture was vortexed for 15 sec and allowed to stand for 30 min at 40 °C for color development. The absorbance was read at 765 nm with a spectrophotometer (UV-1800, Shimadzu, Japan). Total phenolic content was determined as mg of gallic acid equivalent per gram using the equation obtained from a standard gallic acid calibration curve (Figure 1).

## Results

**DPPH free radical scavenging activity**

**Figure 2:**
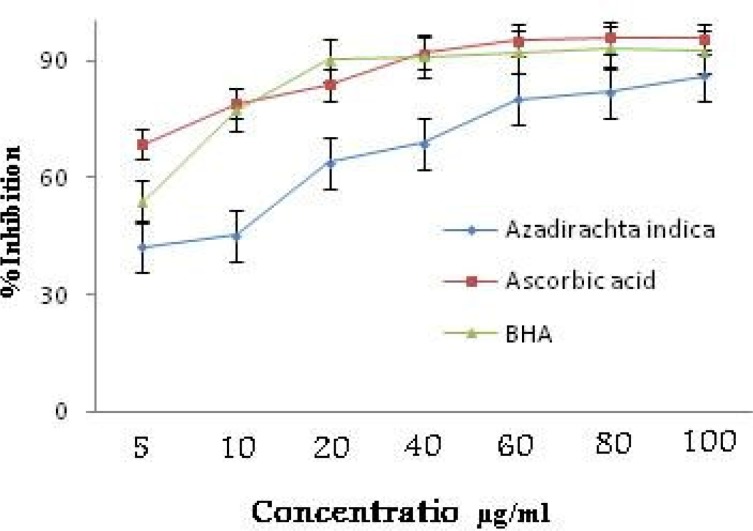
Comparative DPPH radical scavenging activity of ethanol extract of the roots of *Azadirachta indica* and standards of ascorbic acid and butylated hydroxy anisole

The extract showed 86.03±0.04% radical inhibitions at 100 μg/ml whereas at the same concentration the standards ascorbic acid and BHA showed 95.82±0.09 and 93.09±0.06% inhibitions, respectively (Figure 2). The IC_50_ value of extract of *A. indica* was determined as 13.81±0.06 μg/ml where ascorbic acid and BHA showed 2.12±0.02 and 4.87±0.05 μg/ml, respectively. 

**Reducing power assay**

**Figure 3:**
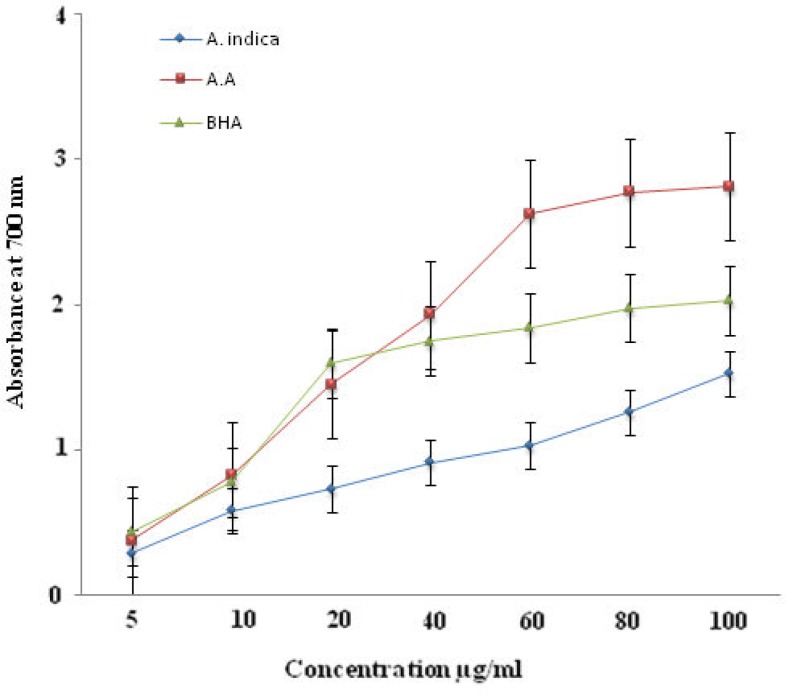
Comparative reducing power assay of ethanol extract of roots of *Azadirachta indica* and standar

Ascorbic acid and BHA were used as positive control for the determination of reducing power of ethanolic extract of *A. indica*. The maximum absorbance for the ethanolic extract of *A. indica *was be 1.523 ±0.026 at 100 μg/ml while that of the standard ascorbic acid and BHA was 2.8111 and 2.031, respectively at the same concentration (Figure 3). 

**Fe** ^++^ **ion chelating ability**

**Figure 4:**
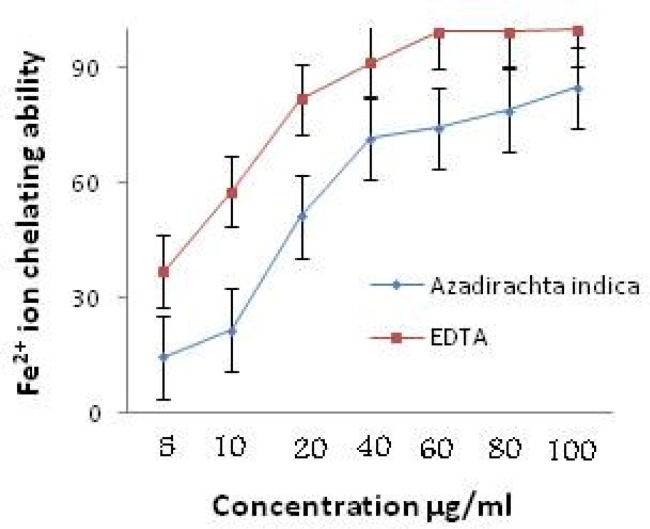
Comparative Fe^2+ ^ion chelating ability of ethanol extract of root of *Azadirachta indica *and standard

Fe^++^ ion chelating ability of ethanol extract of *A. indica* is shown in Figure 4. The extract showed 84.76±0.032% Fe^++^ ion chelating ability at 100 μg/ml where the standard EDTA showed 99.75±0.011% at the same concentration. The IC_50_ value of the extract was also significant while compared with the IC_50_ value of the reference standard EDTA. 

**Table 1:** Total phenolic content determination of ethanolic extract of root of *Azadirachta indica*

**Extract**	**Avg. absorbance at 765 nm**	**Total phenolic content of ethanolic extract of ** ***Azadirachta indica***
**Ethanol extract of ** ***Azadirachta indica***	1.19±0.085	238.81±0.98 mg gallic acid equivalent (GAE) per gm of dry extract

Values are expressed as mean±SD (n=3).

**Total phenolic content**

The amount of total phenolic content was quite high in the ethanolic crude extract of *A. indica* (238.81±0.98 mg/g of gallic acid equivalent) (Table 1). 

## Discussion

DPPH is frequently used to determine radical scavenging activity of natural compounds as it is a stable free radical (Harini et al., 2012 ▶). The present study illustrated that DPPH free radical scavenging activity of *A. indica *increased with the increase of concentration of the extract. In case of reducing power assay, with the increase of concentration, the absorbance of the extract and standards increased gradually. Recently, it has been reported that there is a direct correlation between antioxidant capacities and reducing power of certain plant extracts (Tanaka et al., 1988 ▶).

Fe^++^ ion chelating assay showed that, crude extract and standard compounds interfered with the formation of ferrous and ferrozine complex which suggest that they have chelating activity and are capable of capturing ferrous ion before the formation of ferrozine. Iron is responsible to generate free radicals through the Fenton and Haber–Weiss reaction. However, metal ion chelating activity of an antioxidant compound delayed this oxyradical generation and the consequent oxidative damage. Therefore, metal ion chelating capacity plays a significant role in antioxidant mechanism since it reduces the concentration of the catalyzing transition metal (Amin et al., 2013 ▶).

Phytochemical compounds especially phenolic compounds such as flavonoids, phenyl propanoids, phenolic acids, tannins are very important components for the free radical scavenging and antioxidant activities of plants. Therefore, the relationship between phenolic content of medicinal plants and antioxidant activity is well documented (Amin et al., 2013 ▶; Tanaka et al., 1988 ▶). The result of the present study revealed that the presence of high concentration of phenolic components in the extract may effectively eliminate radicals which contribute directly to antioxidant effect of the system (Duh, 1994 ▶).

In the context, it can be deducted that the crude ethanolic extract of *A. indica *roots contains significant antioxidant activities. However, further researches are suggested to find out responsible constituents and possible active principles for these activities.
